# Accuracy of iodinated contrast and saline delivery using a piston-driven and a peristaltic power injector in CT—an *in vitro* study

**DOI:** 10.1186/s41747-026-00785-0

**Published:** 2026-09-30

**Authors:** Zahra Alyani Nezhad, Aria M. Salyapongse, Courtney J. Goetsch, Rachel M. Bladorn, Carrie M. Bartels, Kelsey L. Schluter, Martin G. Wagner, Giuseppe V. Toia, Timothy P. Szczykutowicz

**Affiliations:** 1 Department of Medical Physics, University of Wisconsin-Madison, Madison, WI USA; 2 Department of Radiology, University of Wisconsin-Madison, Madison, WI USA; 3 Department of Biomedical Engineering, University of Wisconsin-Madison, Madison, WI USA

**Keywords:** Accuracy, CT power injectors, Iodinated contrast media, Piston-based, Peristaltic-based

## Abstract

**Objective:**

To evaluate the accuracy of iodinated contrast media (ICM) and saline flush volumes delivered by a piston- and a peristaltic-based injector in an *in vitro* study.

**Materials and methods:**

Seven clinical injection protocols, ranging from pediatric to bariatric indications and incorporating varying flow rates, ICM volumes, saline volumes, and dilution ratios, were tested. Identical prescriptions were programmed into a piston-based injector and a peristaltic-based injector. Injections were performed into vials. Calibration samples (5–20 mgI/mL) were scanned on a multi-slice CT scanner to generate a calibration curve relating CT number to iodine concentration. The saline test volume and combined ICM + saline flush volume were measured for each injection. CT numbers of mixtures were converted to iodine concentration using the calibration curve, and delivered ICM and saline volumes were calculated. Percent errors between expected and measured volumes were determined. A Wilcoxon signed-rank test assessed statistical significance.

**Results:**

For the peristaltic-based injector, ICM volume error ranged from 4.0% to 10.0% and saline flush error from 0.6% to 9.2%. For the piston-based injector, ICM volume error ranged from 9.9% to 40.0% and saline flush error from 1.5% to 50.2%. Differences between expected and delivered volumes were statistically significant across protocols (*p* = 0.031/= 0.035), except for peristaltic saline flush protocols (Ped 12 mL (*p* = 0.094), Adult 170 mL (*p* = 0.156), and High Flow (*p* = 0.093)).

**Conclusions:**

Both injectors delivered excess ICM while saline flush volumes showed variability. These findings underscore the importance of performance verification to ensure accurate and consistent contrast delivery in clinical practice.

**Key Points:**

***Question*** Automated injectors often delivered more contrast than expected.

***Findings*** Both systems often underdelivered saline with some instances of overdelivery.

***Relevance statement*** Unrecognized variability in contrast and saline delivery across different injection settings may affect enhancement consistency, contrast exposure, and diagnostic reliability. Broader injector performance evaluation across clinically used protocols and clinically simulated setups may improve CT practice.

**Graphical Abstract:**

**Figure:**
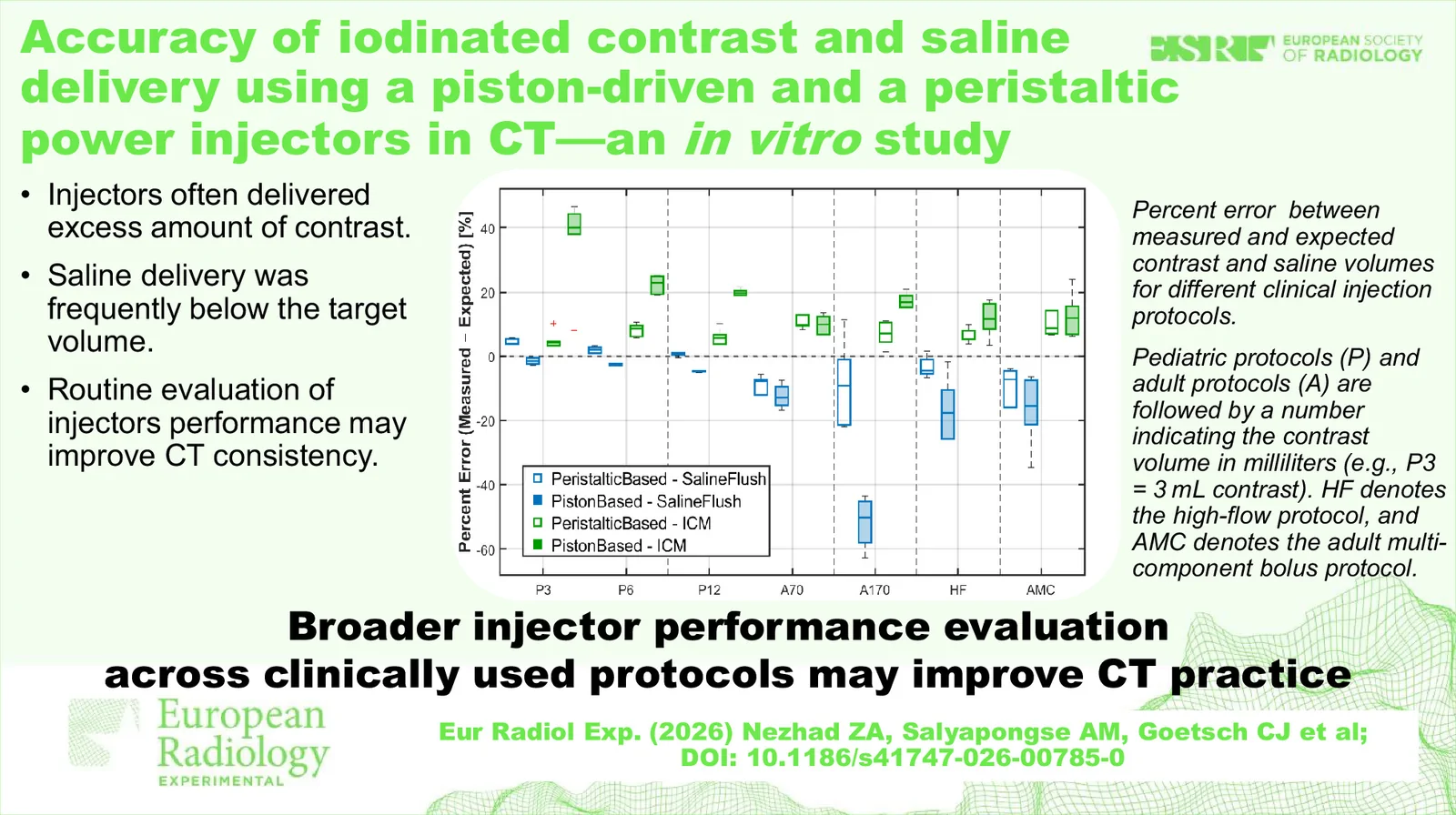

## Background

CT scans use iodinated contrast media (ICM) to enhance soft tissue contrast [1]. Higher ICM concentrations increase viscosity [2], preventing manual injection at clinical flow rates [3]. Automated power injectors overcome these limitations.

Power injectors provide controlled, uniform flow and have been standard in clinical practice for decades [4]. The main types are piston- and peristaltic-based, both using positive-displacement pumps to deliver ICM [4]. Piston injectors push ICM from a syringe *via* a mechanical piston [5], while peristaltic injectors use rollers to advance ICM through tubing [4]. These differences may affect contrast delivery accuracy.

Many factors influence iodine enhancement, including ICM volume, injection flow rate, and saline flush volume [2, 6, 7]. The ICM volume, saline volume, and the flow rate are directly controlled by the power injector’s settings. Hence, it is essential for the power injectors to be accurate to the prescription of ICM and saline solution based on the clinical protocol. There are several studies evaluating the performance of power injectors [4, 8–11]. To the best of our knowledge, limited data exists evaluating the accuracy of ICM and saline volume injected by different power injectors using real clinical ICM protocols in CT.

In this study, we aim to evaluate the accuracy of iodine contrast media and saline flush volumes delivered by two major categories of CT power injectors: piston-based and peristaltic-based systems. The primary objective was to quantify the delivered ICM volume, saline flush volume, and resulting ICM concentration within the patient tubing to identify potential deviations from the prescribed clinical protocol.

## Materials and methods

This study evaluated the accuracy of two contrast injection systems: the piston-based MEDRAD® Stellant CT Injection System (Bayer AG) and the peristaltic-based Ulrich CT motion™ Injector (Ulrich Medical) using clinically established ICM protocols. Protocols reflected routine practice, with varying ICM volumes and flow rates, to quantify delivered ICM and saline flush volumes relative to expected values. Details are in Table 1. Mixed flow injections delivered the main ICM bolus, followed by 50% ICM/50% saline micro-boluses, then the main saline flush.

**Table 1:** Designed clinical contrast administration protocols used for the evaluation of injector systems

Protocol name	Test bolus (mL)	Iohexol 350 mg/mL (mL)	Saline flush (mL)	Flow rate (mL/s)
Peds 3 mL	20	3	50	2
Peds 6 mL	20	6	50	2
Peds 12 mL	20	12	50	2
Adult 70 mL	20	70	50	3
Adult 170 mL	20	170	50	3
High flow	20	85	50	6
Adult, multi-component bolus	30	65 (main bolus), 10 × 3 mL (micro boluses) Total = 95 mL	30 (main saline flush bolus), 10 × 3 mL (micro boluses) Total = 60 mL	5

Pediatric (Peds) protocols (3, 6, and 12 mL) are commonly used for pediatric routine chest/abdomen/pelvis examinations. Adult protocols (70 and 170 mL) are typically used for routine abdomen/pelvis imaging and for high-image-quality (HIQ) cancer follow-up abdomen/pelvis studies for small and bariatric sizes, respectively. The high-flow protocol is used for gated chest examinations. The Adult, multi-component bolus protocol is used for abdominal CT angiography, *e.g*., active bleeder and mesenteric ischemia protocols

We developed a quantitative framework to relate measured CT numbers (Hounsfield units) to iodine concentration under clinically relevant conditions. Calibration curves were first established by scanning iodine concentrations representative of clinical practice. Contrast injections were then performed using standard clinical protocols, and the resulting CT images were analyzed. Measured CT numbers were converted to iodine concentrations using the calibration curves and compared with expected values assuming accurate injection. Each step of this workflow is detailed in the following subsections.

### Calibration standard curve

To enable injector testing, a calibration curve was first established that allowed conversion between a given CT number (measured in Hounsfield units) and a known ICM concentration. A calibration standard was prepared by creating multiple iodine–saline solutions with known concentrations, based on typical clinical iodine concentrations [12] ranging from 5–20 mgI/mL. These solutions were scanned to generate a calibration curve relating CT number to iodine concentration.

Iohexol 350 (Omnipaque® 350 (350 mg I/mL), GE Healthcare) was used as the stock ICM. A 300 mL diluted solution with a concentration of 20 mgI/mL was first prepared using the standard dilution equation (Eq. 1) [13]:

1 $${C}_{{Stock}}{V}_{{stock}}={C}_{{Sol}}{V}_{{Sol}}$$

where ${C}_{{Stock}}$ is the concentration of the stock ICM, ${C}_{{Sol}}$ is the concentration of the diluted solution, and ${V}_{{stock}}$ is the volume of the stock ICM required, ${V}_{{Sol}}$ is the total volume of the diluted solution.

This calculation yielded 17.14 mL of iohexol mixed with 282.86 mL of saline to produce the 20 mgI/mL stock solution. This diluted solution was subsequently used to prepare additional calibration standards with varying iodine concentrations; each adjusted to a final volume of 25 mL. A micropipette was used to ensure precise measurement of both the ICM and saline volumes. The specific volumes of saline and 20 mgI/mL ICM used to prepare each calibration standard are summarized in Table 2. For statistical purposes, two replicates of each standard concentration were prepared.

**Table 2:** Preparation of iodine calibration standards

Standard	Saline (mL)	Diluted contrast (20 mg I/mL) (mL)
20 mg/mL	0	25
15 mg/mL	6.25	18.75
12 mg/mL	10	15
10 mg/mL	12.5	12.5
8 mg/mL	15	10
5 mg/mL	18.75	6.25
0 mg/mL	25	0

A 20 mg I/mL iohexol solution, prepared from stock iohexol (Omnipaque® 350, GE Healthcare), was mixed with saline in specified volumes to generate calibration standards of varying iodine concentrations. All standards were prepared to a final volume of 25 mL using a micropipette, with two replicates per concentration

After preparation of two solutions for each concentration, each solution was scanned using a multi-slice CT scanner (Discovery HD750, GE Healthcare) using the following technique: tube potential of 120 kV, tube current of 70 mA, slice thickness = 2.5 mm, reconstruction kernel = standard (vendor’s soft tissue kernel), pitch = 0.516:1, rotation time = 1 s, collimation = 40 mm. CT numbers were obtained from each vial using a 360 mm² region of interest (ROI) placed at the center of the vial, avoiding edge artifacts. A calibration curve of CT number *versus* iodine concentration was then generated.

### Preparation of injection samples for piston- and peristaltic-based injector protocols

Six replicate injections were prepared for each protocol in Table 1. Large beakers, injector patient kits, a micropipette, saline, and a graduated cylinder were used. Protocols were programmed into both piston- and peristaltic-based injectors. For the tested piston injector, a new patient kit (*i.e*., two piston-based syringes, two spikes, and one long forked patient tube) was used for each injection, while for the peristaltic injector, only the patient line was replaced. A 500 mL beaker was positioned to receive the line output, secured to prevent overflow, and a line integrity test injection was performed and immediately paused.

The injected volume was transferred from the beaker into a graduated cylinder, and the line-integrity test injection volume (V_test_) was recorded. This volume was then poured into a separate, labeled beaker and set aside. The original beaker was repositioned, and the injector line was returned to it. The remaining portion of the injection protocol was then executed. Following completion, the remaining injected volume (V_ICM + flush_) was transferred to a graduated cylinder and recorded. This solution was subsequently combined with the previously collected test injection volume in the reserved beaker. The test/line-integrity (test bolus) volume is part of the injector’s operational protocol and reflects routine device setup used in clinical practice. Hence, we included it in the analytical reconstruction to replicate real-world injection conditions, where this volume is ultimately delivered to the patient as part of the full contrast administration process. The combined mixture was stirred for approximately 1 min, either manually or using an automated magnetic stir bar, to ensure homogeneity.

### Calculation of actual delivered ICM and saline volumes

The standard dilution equation was used for all subsequent calculations. The iodine concentration of each mixture (test injection + ICM + saline flush) for each protocol was first calculated as follows (Eq. (1)):

2 $${C}_{{Mix}}=\frac{{C}_{350{{\rm{mgI}}}/{{\rm{mL}}}}* {V}_{{\mbox{ICM}},{\mbox{expected}}}}{{V}_{{\mbox{Total}}}}$$

where ${C}_{{Mix}}$ is the initial mixture concentration of saline test injection, ICM and saline flush; ${C}_{350{\mbox{mgI}}/{\mbox{mL}}}$ is the stock ICM concentration (*i.e*., 350 mgI/mL); ${V}_{{\mbox{ICM}},{\mbox{expected}}}$ is the expected injected ICM volume specified by the protocol and ${V}_{{\mbox{Total}}}$ is the total protocol-defined volume of saline, ICM, and saline flush. Because the iodine concentrations resulting from these injections exceeded the typical clinical range and would have expected CT values over the limit of 3,071 HU (*i.e*., the CT scanner cannot reconstruct signal values over 3,071 HU), the mixtures were diluted to clinically relevant levels (10 mg I/mL) before analysis. Each mixture was diluted to a final volume of 25 mL with a target concentration of 10 mgI/mL. The required volumes of the mixture ${V}_{{Mix},{needed}}$ and normal saline ${V}_{{Saline}}$ were calculated based on the initial mixture concentration ${C}_{{Mix}}$ to ensure a total solution volume of 25 mL at a concentration of 10 mgI/mL, using Eqs. (3) and (4):

3 $${V}_{{Mix},{needed}}=\frac{10{\mbox{mgI}}/{\mbox{mL}}* 25\,{{\rm{mL}}}}{{C}_{{Mix}}}$$

4 $${V}_{{Saline}}=25-{V}_{{Mix},{needed}}$$

Table 3 lists the calculated initial concentration of each mixture, along with the corresponding mixture and saline volumes required for each experimental protocol to achieve a final concentration of 10 mg I/mL.

**Table 3:** Calculated initial concentration of each injected mixture, along with the required volumes of the original mixture and normal saline to achieve a final concentration of 10 mg I/mL

Protocol name	Initial mixture concentration (mg I/mL)	Amount of original mixture needed (mL)	Amount of normal saline needed (mL)	Final concentration (mgI/mL)
Peds 3 mL	14.384	17.38	7.620*	10
Peds 6 mL	27.632	9.048	15.952	10
Peds 12 mL	51.220	4.881	20.119	10
Adult 70 mL	175.000	1.429	23.571	10
Adult 170 mL	247.917	1.008	23.992	10
High flow	191.936	1.303	23.697	10
Adult, multi-component bolus	179.730	1.391	23.609	10

An asterisk (*) indicates values that were rounded to three decimal places, which reflects the accuracy limit of the micropipette

These diluted samples were then used to evaluate whether the injection protocols produced the expected iodine concentrations. Each 25 mL vial was scanned, and the iodine concentration was determined using the calibration curve. The actual concentration of the mixture measured for each protocol injection ${C}_{{Mix},{Meas}}$ was subsequently calculated as:

5 $${C}_{{Mix},{Meas}}=\frac{{C}_{{Vial},{Meas}}* 25\,{{\rm{mL}}}}{{V}_{{Mix},{needed}}}$$

where ${C}_{{Vial},{Meas}}$ is the concentration of the 25 mL vial determined from CT measurements using a calibration curve.

The actual ICM volume delivered for each protocol ${V}_{{\mbox{ICM}},{\mbox{Meas}}}$ was then calculated using:

6 $${V}_{{\mbox{ICM}},{\mbox{Meas}}}=\frac{{C}_{{Mix},{Meas}}* {V}_{{\mbox{Total}},{\mbox{Meas}}}}{{C}_{350{\mbox{mgI}}/{\mbox{mL}}}}$$

where ${V}_{{\mbox{Total}},{{\mbox{Meas}}}}$ is the total measured volume of the mixture including test saline, ICM, and saline flush.

The total actual saline volume (test + flush) ${V}_{{\mbox{Saline}},{\mbox{Meas}}}$ was determined as:

7 $${V}_{{\mbox{Saline}},{\mbox{Meas}}}={V}_{{\mbox{Total}},{\mbox{Meas}}}-{V}_{{\mbox{ICM}},{{\mbox{Meas}}}}$$

The volume of the saline flush ${V}_{{\mbox{SalineFlush}}}$ was calculated as:

8 $${V}_{{\mbox{SalineFlush}}}={V}_{{\mbox{Saline}},{{\mbox{Meas}}}}{-}V{test}$$

where $V{test}$ is the measured volume of line-integrity testing saline.

Once combined, each 25 mL prepared mixture or dilution was transferred into labeled 25 mL plastic vials. Each vial was labeled with its corresponding protocol ID, vial number (1–6), and injector type, following a consistent labeling scheme.

All vials were then scanned using the same multi-slice CT system and scanning protocols used for the calibration curve to determine the iohexol concentration. Prior to scanning, each vial was thoroughly shaken to ensure homogeneity. The iohexol concentration in each vial was then quantified using the established calibration curve. The measured ICM and saline flush volumes were compared to the expected values for each protocol and injector type to assess consistency and accuracy of the injectors’ performance using Eq. 9:

9 $${{\rm{Percent\; error}}}=\frac{{V}_{{\mbox{ICM}},{\mbox{Meas}}}-{V}_{{\mbox{ICM}},{\mbox{expected}}}\,}{{V}_{{\mbox{ICM}},{\mbox{expected}}}\,}\times 100$$

### Statistical analysis

All statistical analyses were performed using R statistical and computing software, Version 4.5.2, and MATLAB software (version 2021, MathWorks, Inc.). The model performance of the calibration curve was assessed in MATLAB using the coefficient of determination (R²). In addition, 95% confidence intervals (CI) for the regression coefficients and 95% prediction intervals (PI) for individual observations were calculated to quantify calibration uncertainty.

To account for both calibration regression uncertainty and ROI measurement variability, inverse prediction intervals (IPIs) were calculated using the fitted calibration model for each estimated iodine concentration in R. The IPI implementation follows classical inverse regression methodology for linear calibration models as described by Graybill [14]. The IPIs were converted from iodine concentration to uncertainty in ICM and saline flush volumes using Eq. 3–6.

An uncertainty budget was also constructed to quantify additional sources of measurement error arising from experimental procedures. These included graduated cylinder reading error, pipetting error, residual beaker volume, and uncertainty associated with dilution. We then reported the final propagated error reflecting the combined effects of calibration uncertainty, ROI measurement variability, and experimental measurement errors as IPIs in bias measurements for ICM volume and saline flush volume for all protocols.

Data normality was assessed using the Shapiro–Wilk test in R. Since the assumption of normality was rejected for some groups, a non-parametric statistical test was chosen for all cases. This uniform analytical approach was adopted to maintain consistency across all comparisons within this dataset. Accordingly, the Wilcoxon signed-rank test was applied in R to determine whether the actual injected volumes of ICM and saline flush for each protocol differed significantly from the prescribed contrast volumes. This non-parametric approach was selected to provide a conservative and distribution-free evaluation of the data across all outcomes. Statistical significance was defined as *p* < 0.050 for all tests.

## Results

**Fig. 1:**
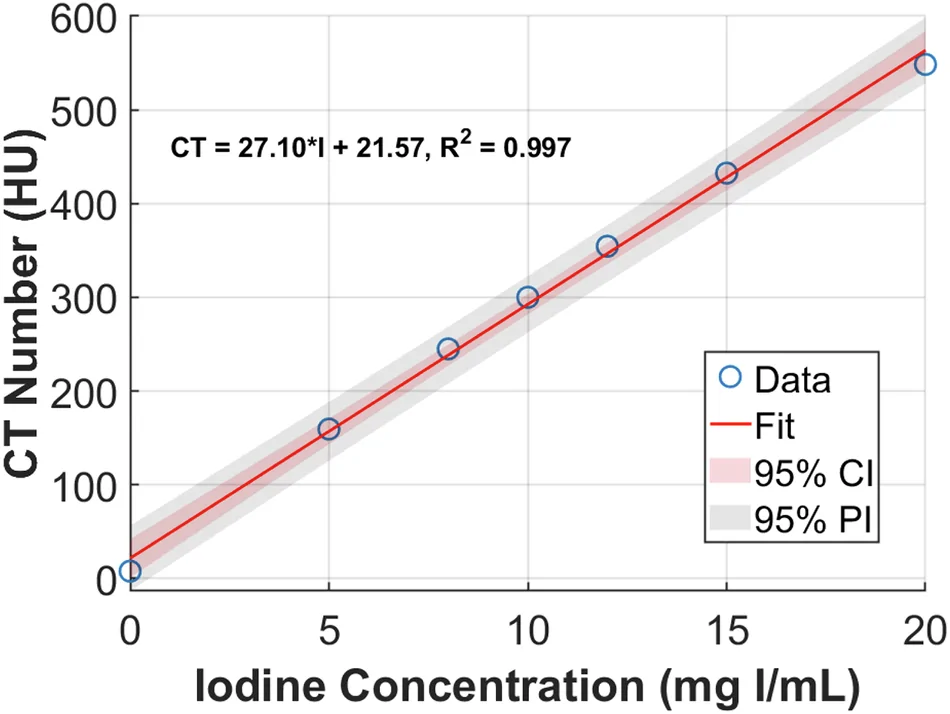
Calibration curve for CT number *versus* iodine concentration. The red line shows the linear fit to the data points with the corresponding equation R^2^ value, 95% confidence interval (CI), and the 95% prediction interval (PI)

Figure 1 shows the CT number *versus* iodine concentration calibration curve. A strong linear relationship was observed between CT number and iodine concentration (R² = 0.997). The fitted regression model demonstrated narrow 95% confidence intervals for the estimated mean response and wider 95% prediction intervals for individual observations, reflecting measurement variability.

**Fig. 2:**
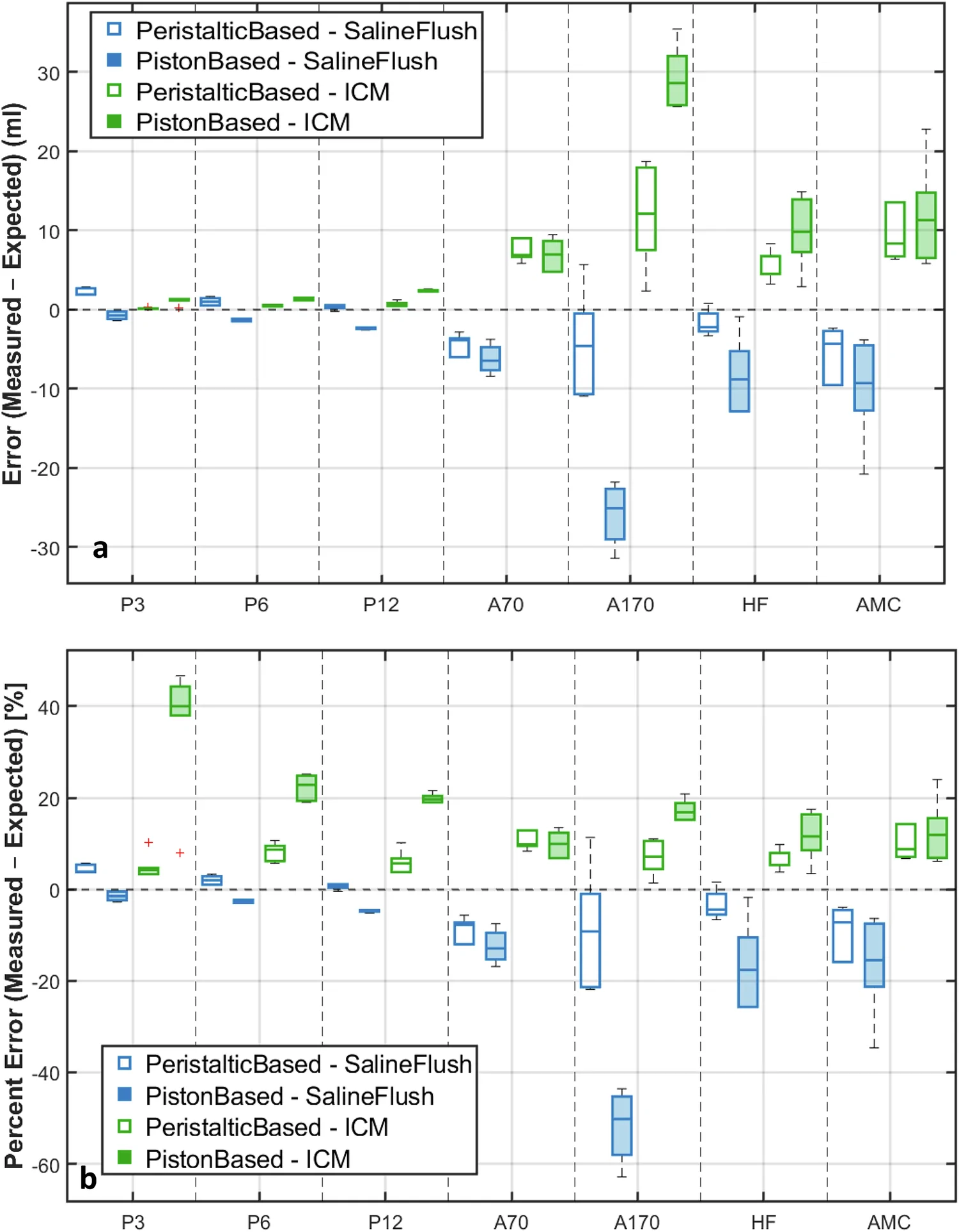
Deviation between expected and measured iodinated contrast media (ICM) and saline flush volumes, shown as (**a**) difference error and (**b**) percent error for each injection protocol, comparing peristaltic-based and piston-based power injectors. In the *x*-axis, pediatric protocols (P) and adult protocols (A) are followed by a number indicating the ICM volume in milliliters defined by that protocol (*e.g*., P3 = 3 mL ICM). HF denotes the high-flow protocol, and AMC denotes the adult multi-component bolus protocol. See Table 1 for a full description of the injection protocols

The median measurement bias, percentage bias, and precision (*i.e*., interquartile range (IQR)) in ICM and saline flush volumes for each protocol using the tested piston-based and peristaltic-based injectors are presented in Fig. 2 and Table 4.

**Table 4:** Median measurement bias and precision (*i.e*., interquartile range (IQR)) were reported for iodine contrast medium (ICM) and saline flush volumes for the tested injectors

Protocol name	Peristaltic-based error of ICM volume (mL) (percent error)	Peristaltic-based error of saline flush volume (mL) (percent error)	Piston-based error of ICM volume (mL) (percent error)	Piston-based error of saline flush volume (mL) (percent error)
Peds 3 mL	0.12 (4.0%) [IPI = 0.6; 0.31] [IQR = 0.03] *p* = 0.035	1.91 (3.8%) [IPI = 2.70; 2.94] [IQR = 0.61] *p* = 0.031	1.20 (40.0%) [IPI = 1.01; 1.29] [IQR = 0.20] *p* = 0.031	-0.74 (1.5%) [IPI = -1.29; -1.01] [IQR = 0.94] *p* = 0.031
Peds 6 mL	0.52 (8.7%) [IPI = 0.24; 0.72] [IQR = 0.17] *p* = 0.031	0.99 (2.0%) [IPI = 0.27; 0.76] [IQR = 0.87] *p* = 0.031	1.37 (22.8%) [IPI = 1.11; 1.61] [IQR = 0.25] *p* = 0.031	-1.37 (2.7%) [IPI = -1.61; -1.11] [IQR = 0.25] *p* = 0.031
Peds 12 mL	0.68 (5.7%) [IPI = 0.35; 1.32] [IQR = 0.31] *p* = 0.031	0.32* (0.6%) [IPI = -0.33; 0.65] [IQR = 0.31] *p* = 0.094	2.36 (20.0%) [IPI = 1.91; 2.90] [IQR = 0.13] *p* = 0.035	-2.36 (4.7%) [IPI = -2.90; -1.91] [IQR = 0.13] *p* = 0.035
Adult 70 mL	6.90 (10.0%) [IPI = 5.15; 10.90] [IQR = 1.80] *p* = 0.035	-3.89 (8.0%) [IPI = -7.90; -2.15] [IQR = 1.80] *p* = 0.035	6.96 (9.9%) [IPI = 5.34; 10.98] [IQR = 3.10] *p* = 0.031	-6.46 (13.0%) [IPI = -9.98; -4.34] [IQR = 2.62] *p* = 0.031
Adult 170 mL	12.10 (7.1%) [IPI = 9.09; 23.08] [IQR = 9.10] *p* = 0.031	-4.60* (9.2%) [IPI = -16.09; -2.09] [IQR = 9.20] *p* = 0.156	28.60 (16.8%) [IPI = 25.79; 39.85] [IQR = 5.42] *p* = 0.031	-25.10 (50.2%) [IPI = -36.85; -22.79] [IQR = 5.80] *p* = 0.031
High flow	6.72 (7.9%) [IPI = 4.85; 11.82] [IQR = 1.71] *p* = 0.035	-2.22* (4.4%) [IPI = -6.82; 0.15] [IQR = 1.96] *p* = 0.093	9.82 (11.5%) [IPI = 1.29; 8.04] [IQR = 5.93] *p* = 0.031	-8.82 (17.6%) [IPI = -7.04; -0.29] [IQR = 6.64] *p* = 0.031
Adult, multi-component bolus	8.33 (8.8%) [IPI = 5.39; 13.10] [IQR = 5.39] *p* = 0.035	-4.33 (7.2%) [IPI = -9.1; -1.4] [IQR = 5.39] *p* = 0.035	11.30 (12.0%) [IPI = 7.84; 15.52] [IQR = 7.15] *p* = 0.031	-9.29 (15.5%) [IPI = -13.53; -5.84] [IQR = 7.15] *p* = 0.031

Uncertainty is reported as inverse prediction interval (IPI). Differences between injected and expected volumes were statistically significant for both injectors, except for protocols marked with (*), which indicate non-significant differences; the corresponding *p*-values are reported

The experimental uncertainty budget included graduated cylinder reading error (± 0.003 mL), pipetting error (± 0.003 mL), residual beaker volume (± 0.003 mL), and protocol-dependent dilution uncertainty (Peds3, Peds6, and Peds12: ± 0.003 mL; Adult70, High flow, and Adult, Multi-component bolus: ± 0.006 mL; Adult170: ± 0.009 mL). When combined using the root-sum-of-squares method, the resulting total experimental volume uncertainties were ± 0.006 mL for Peds3, Peds6, and Peds12, ± 0.008 mL for Adult70, High flow, and Multi-component bolus, and ± 0.010 mL for the Adult170 protocol. Because the experimental uncertainty was negligible relative to the IPIs, the final uncertainty was dominated by the IPIs and is therefore reported as the IPI of ICM volume and saline flush volume in Table 4.

For the peristaltic-based injector, the highest percent error in ICM volume occurred with the Adult 70 mL protocol, where an excess of 6.90 mL of ICM was injected. The most accurate injection was observed in the Pediatric 3 mL protocol, with an additional ICM volume of 0.12 mL, corresponding to a percent bias of 4.0%.

For the tested piston-based injector, the highest percent error in ICM volume was observed in the Pediatric 3 mL protocol, where an excess of 1.20 mL resulted in a 40.0% bias. The most accurate injection occurred in the Adult 70 mL protocol, where an additional 6.96 mL of ICM was injected, corresponding to a 9.9% bias.

For the tested peristaltic-based injector saline flush, the highest percent error occurred in the Adult 170 mL protocol, where a deficit of 4.60 mL resulted in a 9.2% bias. The most accurate saline flush injection was observed in the Ped 12 mL protocol, with an excess of 0.32 mL corresponding to a 0.6% bias.

For the tested piston-based injector saline flush, the highest percent error occurred with the Adult 170 mL protocol, where a deficit of 25.10 mL was injected, resulting in a 50.2% bias. The most accurate saline flush injection was found in the Ped 3 mL protocol, with a deficit of 0.74 mL corresponding to a 1.5% bias.

Wilcoxon signed-rank tests revealed significant differences between injected and expected ICM and saline volumes across most protocols (*p* = 0.031/*p* = 0.035), except for peristaltic saline in Ped 12 mL (*p* = 0.094), Adult 170 mL (*p* = 0.156), and High-Flow protocols (*p* = 0.093).

## Discussion

While vendors are required to submit regulatory data on accuracy and consistency to federal agencies, our understanding is that only a limited number of injection volumes and rates are typically included in these submissions. This study aimed to assess a wider range of volume and rate combinations in light of this. The practice of only testing a few operating points limits the generalizability of these accuracy and consistency tests, leaving the possibility of variance among different injector volume and rate combinations that are not assessed for accuracy or consistency. In this study, we evaluated two types of power injectors (piston-and peristaltic-based) to quantify the delivered volumes of ICM and saline flush, as well as the ICM concentration delivered through the patient tubing, and to assess any deviations from the expected clinical protocol. Delivered volumes of ICM and saline flush were significantly different from expected values across the majority of pediatric and adult protocols for both injector types (*p* = 0.030).

Chaya et al evaluated the same models of injectors used in this study, focusing on the maximum achievable and steady-state flow rates [4]. Their results demonstrated that the tested piston-based injector achieved higher maximum and steady-state flow rates compared to the tested peristaltic-based injector. Because catheter injection into a vial was not performed in our setup, achieving a 5 mL/s flow rate should not have been problematic according to the results from Chaya et al Our findings indicate that, despite the superior flow rate consistency of the piston-based injector reported by Chaya et al, it delivers ICM less accurately than the peristaltic-based injector. However, they did not assess the accuracy of the injected ICM for either injector type.

According to Bae et al, the injected ICM volume equals the product of flow rate and injection duration [2]. Prior work by Chaya et al [4] reported highly consistent flow rates for piston-based injectors. In the absence of direct measurements of individual system components in the present study, the source of the observed volume discrepancies cannot be definitively determined. However, potential contributing factors may include injector variability in injection timing or inaccuracies in the volume drawn into the syringe. These possibilities remain speculative and warrant direct investigation in future studies.

Furthermore, as only two contrast concentrations (300 mg I/mL and 370 mg I/mL) were used by Chaya et al, it remains possible that the reported consistency in flow rate may not hold at other concentrations.

McDermott et al investigated the iodine delivery rate (IDR) and corresponding vascular enhancement achieved with different power injectors [10]. They reported that piston-based injectors produced significantly higher peak vascular enhancement (up to a 48% increase) compared with peristaltic-based injectors programmed for IDRs of 1.8–2.4 gI/s. This difference was attributed to the inability of peristaltic injectors to reach their programmed maximum IDRs. Furthermore, even when both injector types operated within their achievable IDRs, piston-based injectors continued to yield greater enhancement, which the authors attributed to their superior bolus shape and consistency.

McDermott et al reported that the piston-based injector exhibited a higher percent error of up to -2.5% in volume accuracy, whereas the peristaltic-based injector showed a smaller error of approximately 1% [10]. These findings align with our results, where the piston-based injector demonstrated a higher percent error than the peristaltic-based injector. However, in our study, both tested injectors delivered slightly higher ICM volumes than expected, likely due to differences in experimental methods and injection protocols. Specifically, McDermott et al assessed volume accuracy by measuring the output flow rate from the catheter using injection volumes of 40 mL and 54 mL at flow rates of 4.1 mL/s and 5.4 mL/s, respectively [10]. In contrast, our study encompassed a broader range of injection volumes representative of both pediatric and adult protocols, with flow rates of 2, 3, 5, and 6 mL/s. The percent difference in ICM volume injected could explain why McDermott et al observed higher vascular enhancement with piston-based injectors.

In our study, even at the highest flow rate of 6 mL/s used in the high-flow protocol, the resulting IDR was 2.1 gI/s, which is within the achievable range reported by McDermott et al and Chaya et al [4, 10] for both Medrad and Ulrich injectors. Therefore, the effect of IDR limitation is not expected in our results. Although McDermot et al [6] mentioned that automated filing of piston-based injectors results in zero ICM volume delivery error compared to under-delivery of ICM in a manual technique, our study shows that the piston-based injector is delivering more ICM than expected. However, our study did not evaluate the errors in ICM delivery volume with a manual technique.

Figure 8 from Bae et al [2], which describes the relationship between ICM volume and hepatic enhancement, was used to derive the equation: hepatic enhancement change = 0.008 × volume + 0.165. Based on this relationship, a 16.8% bias in ICM volume with a piston-based adult 170 mL protocol corresponds to approximately a 15% change in enhancement magnitude. In this setting, even minor absolute deviations in delivered ICM volume can result in meaningful changes in contrast enhancement. However, very small deviations may not always be clinically significant. While radiology does not have an accepted threshold for medical error in terms of drug dosage misadministration, many clinical studies define a dosing error as a deviation from planned drug delivery with consideration of the type of drug and patient weight [15].

For example, early termination of an injection in a pediatric case can lead to a substantial reduction in the delivered ICM volume and consequently reduced vascular enhancement. In clinical practice, injection-related volume delivery errors may not be explicitly recognized. As a result, sites may empirically adjust their ICM protocols over time to achieve acceptable image quality, implicitly compensating for these systematic errors. When such empirically optimized protocols are shared across institutions or applied after injector replacement, the same protocols may yield different enhancement outcomes if the new injector systems exhibit different systematic delivery errors.

Moreover, it is worth noting that, although the Adult 170 mL and High Flow peristaltic protocols showed no statistically significant saline flush differences, the magnitude and variability of errors could still have a clinical impact.

There are some limitations to this study. Firstly, only a limited number of commonly used clinical protocols were evaluated for injection accuracy. Further investigations could be conducted if any clinical issues arise for specific protocols. Additionally, the experiments were performed using a beaker setup without a catheter or human tissue simulation. In clinical practice, an inappropriate catheter size could restrict the achievable flow rate, potentially causing greater deviations between the expected and delivered ICM and saline flush volumes. This study is also limited by the relatively small sample size (*n* = 6), which limits the statistical power. Consequently, non-significant results, including those observed for the saline flush, should be interpreted cautiously, as the study may have been underpowered to detect meaningful differences. Additionally, only a single unit from each injector type was evaluated (one MEDRAD Stellant and one Ulrich CT Motion system). As a result, the observed differences may reflect unit-specific factors such as device wear, calibration, or manufacturing variability, rather than inherent differences between injector designs. Therefore, our findings should be interpreted as comparisons between the tested units rather than definitive comparisons between injector categories. Future studies including multiple units per injector type are needed to assess inter-device variability and confirm generalizability. Lastly, a subset of our clinical scanners utilizes contrast warmers. This study was performed using room temperature contrast; we expect the results to change with contrast temperature.

In conclusion, in this bench study, both injectors delivered excess ICM while saline flush volumes showed variability, with a tendency toward under-delivery in most cases. Our findings indicate that the tested piston-based injector demonstrated higher delivery error compared to the tested peristaltic-based injector, suggesting lower performance under the evaluated conditions. These results indicate system-level differences in delivery performance under controlled conditions and suggest the need for further evaluation in clinically simulated or *in vivo* settings. These inaccuracies may affect enhancement consistency and contrast dosing. Routine performance verification across clinically relevant injection settings is recommended to ensure accurate and reliable contrast delivery in CT practice.

## Supplementary information

Supplementary information

## Data Availability

The datasets generated during and/or analyzed during the current study are not publicly available, but are available from the corresponding author on reasonable request.

## Abbreviations

CT: Computed tomography
ICM: Iodinated contrast media
