# Supplementary material for: Accuracy of iodinated contrast and saline delivery using a piston-driven and a peristaltic power injector in CT—an in vitro study
Source: Eur Radiol Exp. 2026 Sep 30;10:143. doi: 10.1186/s41747-026-00785-0 (PMC13627536; doi:10.1186/s41747-026-00785-0)
Supplement: Supplementary file 1 [file 41747_2026_785_MOESM1_ESM.pdf]

Accuracy of Iodinated Contrast and Saline Delivery Using a Piston-Driven and a Peristaltic Power Injectors in CT-An in vitro study

ELECTRONIC SUPPLEMENTARY MATERIAL

**calibration vials**	
Conc(mgI/mL)	Ctnumber (ROI area 360 mm2, 71 mm perim)
20	547
20	550
15	433
15	432
12	355
12	354
10	301
10	299
8	244
8	246
5	159
5	160
0	7.54
0	8.05

Ulrich					
				**Measured volumes**	
		**CT number**		**test**	**Contrast+flush**
A1	Ped 3ml	305		21	56
A2	Ped 3ml	290		21	56
A3	Ped 3ml	292		21	55
A4	Ped 3ml	293		21	55
A5	Ped 3ml	290		21	55
A6	Ped 3ml	289		21	55
C1	Ped 12ml	313		21	63
C2	Ped 12ml	299		21	63
C3	Ped 12ml	303		21	63
C4	Ped 12ml	296		21	63
C5	Ped 12ml	304		21	63
C6	Ped 12ml	299		20	63
G1	Adult 170 ml	304		21	227
G2	Adult 170 ml	295		21	228
G3	Adult 170 ml	284		21	228
G4	Adult 170 ml	309		21	228
G5	Adult 170 ml	293		21	227
G6	Adult 170 ml	309		21	227
B1	Ped 6ml	310		21	57
B2	Ped 6ml	302		21	57
B3	Ped 6ml	310		21	58
B4	Ped 6ml	297		21	58
B5	Ped 6ml	307		21	58
B6	Ped 6ml	307		21	57
H1	High flow	303		21	140
H2	High flow	308		21	140
H3	High flow	305		21	139
H4	High flow	305		21	139
H5	High flow	294		21	139
H6	High flow	298		21	139
J1	Mixed Adult	310		31	159
J2	Mixed Adult	307		31	159
J3	Mixed Adult	323		31	159
J4	Mixed Adult	303		31	159
J5	Mixed Adult	323		31	159
J6	Mixed Adult	304		31	159
D1	Adult 70ml	310		21	123
D2	Adult 70ml	319		21	123
D3	Adult 70ml	311		21	123
D4	Adult 70ml	307		21	123
D5	Adult 70ml	319		21	123
D6	Adult 70ml	311		21	123

Medrad					
A1	Ped 3ml	419		20	53
A2	Ped 3ml	314		20	53
A3	Ped 3ml	396		20	53
A4	Ped 3ml	408		20	54
A5	Ped 3ml	403		20	53
A6	Ped 3ml	394		20	54
B1	Ped 6ml	361		20	56
B2	Ped 6ml	355		20	56
B3	Ped 6ml	354		20	56
B4	Ped 6ml	345		20	56
B5	Ped 6ml	344		20	56
B6	Ped 6ml	360		20	56
C1	Ped 12ml	344		20	62
C2	Ped 12ml	346		20	62
C3	Ped 12ml	344		20	62
C4	Ped 12ml	351		20	62
C5	Ped 12ml	346		20	62
C6	Ped 12ml	348		20	62
D1	Adult 70 ml	327		20	121
D2	Adult 70 ml	311		20	120
D3	Adult 70 ml	321		20	120
D4	Adult 70 ml	309		20	121
D5	Adult 70 ml	324		20	121
D6	Adult 70 ml	316		20	121
G1	Adult 170 ml	337		20	223
G2	Adult 170 ml	334		20	223
G3	Adult 170 ml	328		20	224
G4	Adult 170 ml	327		20	223
G5	Adult 170 ml	326		20	224
G6	Adult 170 ml	341		20	224
H1	High flow	335		20	136
H2	High flow	312		20	137
H3	High flow	316		20	136
H4	High flow	300		19	137
H5	High flow	328		20	136
H6	High flow	336		20	137
J1	Mixed Adult	354		30	157
J2	Mixed Adult	308		30	157
J3	Mixed Adult	333		29	157
J4	Mixed Adult	327		30	156
J5	Mixed Adult	316		30	157
J6	Mixed Adult	306		30	157
